# Therapeutic modulation of energy metabolism in ischemic brain injury

**DOI:** 10.4103/NRR.NRR-D-25-00592

**Published:** 2025-09-29

**Authors:** Egor Y. Plotnikov, Nadezda V. Andrianova

**Affiliations:** A.N. Belozersky Institute of Physico-Chemical Biology, Lomonosov Moscow State University, Moscow, Russia

Acute cerebral ischemia caused by stroke, traumatic brain injury (TBI), or systemic acute conditions such as hemorrhagic shock, cardiac arrest, or disseminated intravascular coagulation results in an energy crisis in local sites or the whole brain. The disruption of cerebral blood flow deprives the brain cells of oxygen and glucose, the essential substrates for adenosine triphosphate (ATP) synthesis. As a result, oxidative phosphorylation in the mitochondria fails, forcing cells to rely on anaerobic glycolysis (He et al., 2020). Although this compensatory mechanism maintains short-term energy production under hypoxic conditions, overall ATP production is significantly reduced. Neurons, which are highly susceptible to ischemic injury, deplete their ATP stores faster than glial cells (e.g., astrocytes), which have some energy reserves. This energy deficit disrupts ion pump activity, impairs membrane potential homeostasis, and triggers a cascade of pathologic events, including excessive release of excitatory neurotransmitters (e.g., glutamate) and intracellular Ca²⁺ overload that initiates neuronal death. At the same time, impaired mitochondrial metabolism exacerbates the production of reactive oxygen species (ROS) and increases oxidative stress (Yang et al., 2018). Under hypoxic conditions, glycolysis is upregulated but cannot balance the brain’s energy needs and promotes lactic acid accumulation and tissue acidosis. Lactic acidosis in combination with dysregulation of ion concentrations (e.g., imbalance of H⁺, Na⁺, and Ca²⁺) further injures neuronal excitability and exacerbates cell damage.

**Strategies for metabolic correction of brain damage:** The promising direction for the treatment and prevention of the consequences of acute brain disorders is to influence cell metabolism. Such therapeutic strategies aim not only to restore blood flow, but also to correct metabolic imbalances, protect mitochondrial integrity and replenish cellular energy stores. Various alternative metabolic substrates are being tested in experimental models and clinical trials to modulate cerebral energy metabolism.

In particular, the use of tricarboxylic acid cycle intermediates bypasses the blockades in aerobic metabolism and provides mitochondria with substrates for oxidative phosphorylation and synthetic processes. The use of such metabolites reduces dependence on anaerobic glycolysis and decreases lactate accumulation (Xie et al., 2022). Experimental studies in models of cerebral ischemia, TBI or hemorrhagic shock have shown that infusion of tricarboxylic acid cycle metabolites (such as pyruvate and fumarate) restores ATP levels in the brain, reduces tissue damage and inflammatory responses, and improves neurological outcomes. However, it should be noted that these metabolites have a relatively low bioavailability, so it is necessary to develop derivatives that penetrate better into the cells and cross the blood–brain barrier (BBB). To overcome these limitations, prodrugs of tricarboxylic acid cycle intermediates (e.g., dimethyl succinate, diethyl succinate, dicholine succinate, and dimethyl fumarate) are being developed for better membrane permeability. Through esterification and the associated masking of polar carboxylic acid groups, these compounds gain lipophilicity, which improves cellular uptake and penetration of the BBB for targeted metabolic therapy.

One of the promising methods for treating acute injuries is oxidative phosphorylation bypass, which refers to alternative pathways that obtain metabolic substrates for the tricarboxylic acid cycle or allow electrons to circumvent blocked or dysfunctional mitochondrial respiratory chain complexes (I–IV). The use of methylene blue, an alternative electron carrier that bypasses the blockages in complexes I and III of the respiratory chain, maintains ATP synthesis even under hypoxic conditions, but carries the risk of hemolysis at high doses. Coenzyme Q10, a component of the respiratory chain, improves electron transfer and neutralizes ROS. Due to these properties, it reduces the area of damage in models of cerebral ischemia. However, similar to the metabolites of the tricarboxylic acid cycle and creatine, coenzyme Q10 has a rather low bioavailability, although it can be improved by analogs such as coenzyme Q4. The first clinically tested drug for mitochondrial bypass was vitamin K3 (menadione) in combination with ascorbate, which successfully restored electron transport in patients with complex III deficiency, as evidenced by a 21-fold increase in phosphocreatine recovery rate and improved muscle function. A similar mechanism is assumed for vitamin K2 (MK-7 and MK-4), which acts as an endogenous electron carrier to complex III that restores mitochondrial function in neurodegeneration. One more approach, glycerol-3-phosphate-dependent shuttle supports ATP production by feeding electrons from glycolysis into the ubiquinone pool via mitochondrial G3P dehydrogenase, bypassing Crebs cycle and complex I.

Targeting the mitochondria is a promising approach to correct metabolism, as mitochondria play a key role in the pathogenesis of ischemic brain damage. The neuroprotective properties of the peptide SS-31, which stabilizes the inner mitochondrial membrane by selective binding to cardiolipin, improves respiratory chain function and reduces ROS production, have been demonstrated. One way to improve the bioavailability and efficacy of compounds such as coenzyme Q10 and other antioxidants is to target them to the mitochondria using hydrophobic cations such as triphenyl-phosphonium. These strategies have led to the development of mitochondria-targeted antioxidants, e.g., MitoQ, SkQ, and mitoTEMPO, which are also effective in ischemic brain pathologies.

Another approach to improve energy metabolism in the brain is to increase creatine levels, which maintains energy homeostasis via the creatine-phosphocreatine shunt and rapidly regenerates ATP by serving as an ATP buffer when energy is depleted. Animal studies have shown that creatine administration reduces stroke volume and improves motor function. However, ingestion of exogenous creatine can lead to increased tissue water, gastrointestinal discomfort, and dehydration. While creatine is generally safe when used correctly, its clinical efficacy needs to be further validated, particularly in relation to BBB penetration.

Systemic approaches, such as diets, are also used to modulate energy metabolism. Caloric restriction prior to stroke onset is an effective strategy for neuroprotection, improving mitochondrial quality control, antioxidant defense, autophagy, sirtuin activity, neurotrophic support, and chaperone function while reducing inflammation and oxidative stress. Body temperature also has a strong influence on metabolic processes in the tissue. In particular, therapeutic hypothermia slows down the metabolism, reduces the cells’ ATP requirements, and suppresses ROS production. Clinical studies have shown that hypothermia improves the survival of cardiac arrest patients and reduces neurological deficits in stroke patients. However, this technique must be closely monitored due to the risk of cardiac arrhythmias, infections, and other complications.

Another approach to metabolic correction in acute brain injury that deserves closer consideration is the use of ketone bodies. Given the limitations and side effects of some other metabolic correction strategies, ketone bodies are considered promising therapeutics for acute brain injury due to their ability to shift energy supply from glycolysis and support mitochondrial energy production.

**Ketone bodies as therapeutic agents in brain damage:** Ketone bodies, in particular β-hydroxybutyrate, acetoacetate, and acetone, serve as an alternative energy source in glucose deficiency (Arora et al., 2024). When glucose metabolism is impaired, the brain resorts to ketone bodies as an alternative energy source to maintain neuronal function. These substrates are oxidized by the mitochondria, bypassing the glycolytic pathway, to enable ATP synthesis, which is particularly important during ischemia when glucose availability is reduced (Kolb et al., 2021). Indeed, ketone bodies and the ketogenic diet have shown a neuroprotective effect in ischemic conditions by improving energy metabolism, reducing free radical formation, and suppressing inflammatory processes (Jang et al., 2023).

Ketone bodies can be administered exogenously or produced endogenously under certain diets. One of the most used approaches to increase blood ketone body levels is the ketogenic diet (KD), in which carbohydrate intake is strictly limited and fat intake is increased. The KD leads to a shift in metabolism from glycolysis to oxidative phosphorylation. Similarly, exercise and fasting or caloric restriction result in increased formation of ketone bodies, reducing the availability of glucose, lowering insulin levels, and promoting lipolysis, leading to ketogenesis in the liver, where fatty acids are converted to ketone bodies that are used as alternative fuels in distant tissues. KD was originally used to treat epilepsy, as ketone bodies promote the recovery of neuronal function and reduce seizures. Further studies have shown that KD can influence mitochondrial function, improve some pathological processes such as inflammation and oxidative stress, and participate in epigenetic regulation. Following the discovery of neuroprotective effects, KD has been proposed as a potential therapy to prevent ischemic and traumatic brain injury or to promote recovery (Gough et al., 2021).

Similar neuroprotective effects have been demonstrated with the administration of exogenous ketone bodies. For example, treatment with β-hydroxybutyrate, 1,3-butanediol or acetoacetate improved ischemic stroke and TBI in animal models. In both the acute and chronic phases of brain injury, mitochondrial integrity was severely compromised, resulting in impaired ATP production and triggering mitochondria-dependent cell death, including apoptosis, while ketones restored energy supply, inhibited cell death, and stimulated brain-derived neurotrophic factor synthesis, thereby promoting neuronal recovery. In clinical studies, oral ketone supplements normalize neurological parameters and metabolic markers in stroke patients. The beneficial effects of ketone bodies today include restoration of energy supply and mitochondrial function as well as anti-inflammatory responses, attenuation of oxidative stress, epigenetic regulation, and inhibition of mitochondrial permeability transition (Makievskaya et al., 2023).

KD and ketone bodies influence several important signaling pathways (Jang et al., 2023). Thus, KD upregulates cytoprotective signaling pathways such as stabilization of hypoxia-inducible factor 1 α, protein kinase B (AKT) phosphorylation, and AMP-activated protein kinase activation, enhancing postischemic metabolic recovery. KD has been shown to suppress the NLR family pyrin domain containing 3 (NLRP3) inflammasome by inhibiting endoplasmic reticulum stress and Drp1-mediated mitochondrial fission, reducing ROS formation, and suppressing the ROS-NLRP3 axis. The anti-inflammatory effects of β-hydroxybutyrate can be mediated by activating the hydroxycarboxylic acid receptor 2 receptor on immune cells, thereby inhibiting nuclear factor κB translocation, suppressing the production of pro-inflammatory cytokines, and modulating macrophage polarization towards an anti-inflammatory phenotype (Graff et al., 2016). β-Hydroxybutyrate mediates epigenetic regulation via inhibition of HDAC I/II and promotes FoxO-dependent transcription. Finally, KD counteracts insulin resistance by recovering PI3K/AKT/hypoxia-inducible factor 1 α signaling and exerts neuromodulatory effects by inhibiting the mammalian target of rapamycin pathway.

**Problems with the implementation of KD and ketone bodies in clinical practice:** Despite their promising effects, many strategies for correcting metabolism are associated with challenges. Some problems have already been described, and in the following section, we will look at the difficulties in implementing KD and ketone bodies in clinical practice. Despite the neuroprotective effects of KD and ketone bodies demonstrated in some studies, there are certain papers in which these approaches to metabolic correction either have no effect on the severity of the damage or even exacerbate it (Makievskaya et al., 2023). It seems that the effectiveness of the use of KD and its mimetics in the experimental studies depends on the stroke model, the animal species, as well as the concomitant diseases and the metabolic state of the organism.

**Figure 1:**
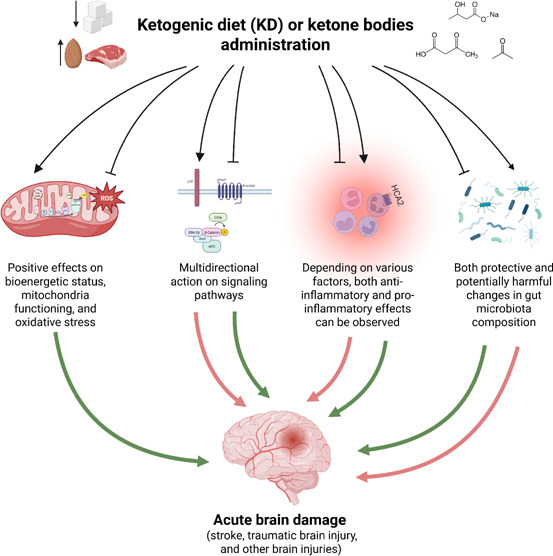
Multidirectional effect of KD or exogenous ketone bodies on the brain in stroke, TBI, and other brain injuries. KD and ketone bodies have a positive effect on mitochondria and energy supply (marked with a green arrow). However, the effects on intracellular signaling and immune response may be unidirectional with stroke-induced changes and could therefore be considered negative (marked with red arrows). KD and its mimetics increase the composition of both bacteria with beneficial properties and those associated with certain pathologies, so the implications of such an effect need to be further clarified. Created with BioRender.com.

Stroke negatively affects the functioning of almost all systems of neural cells and alters the expression of many thousands of genes; similar changes are observed in the brain in TBI and other critical brain diseases. As shown by a comprehensive analysis of the transcriptome, metabolome, and microbiome, KD has also significantly affected gene expression in the brain, metabolites in the blood and the composition of the gut microbiota (Zharikova et al., 2025). Some of the KD-induced changes can be interpreted as positive, while others are unidirectional with the changes caused by the stroke and could therefore be considered negative (**Figure 1**). For example, the KD is known to have a positive effect on tissue metabolism by causing a metabolic shift (Puchalska and Crawford, 2021). Indeed, a large-scale study of the metabolome and transcriptome in KD showed increased levels of ketone bodies, long-chain fatty acids, medium- and long-chain acylcarnitines, and decreased levels of short-chain acylcarnitines and some amino acids, as well as altered expression of genes involved in glucose and fatty acid metabolism, mitochondrial function, mitochondrial protein transport and the electron transport chain (Zharikova et al., 2025). However, the KD had an ambiguous effect on some intracellular signaling pathways, such as Wnt-associated signaling. Thus, KD increased the expression of about 50 participants of this signaling pathway, while it decreased the expression of another 30 genes, and only 13 genes changed in the opposite manner to the middle cerebral artery occlusion-induced changes. Although KD is thought to have more of an anti-inflammatory effect (Monda et al., 2024), this diet can upregulate the expression of proinflammatory cytokines and increase markers of immune and activated glial cells during stroke. Similarly, the KD could result in both increased levels of phospho-AKT in brain tissue and in a decrease of AKT phosphorylation (McDaniel et al., 2011). In addition, KD has been shown to induce ROS production, but this can activate the Nrf2 signaling, leading to permanent upregulation of antioxidant enzymes (Miller et al., 2010). Finally, while KD-mediated inhibition of the mitochondrial permeability transition offers cytoprotective benefits, it may carry the inherent risk of hindering apoptosis-mediated elimination of compromised cells.

Similarly, the KD has a significant impact on the gut microbiota, leading to enrichment with both beneficial bacteria and those associated with certain pathologies. For instance, a 2-month KD significantly increased the abundance of the *Ruminococcaceae* family within the gut microbiome (Zharikova et al., 2025). This shift is potentially detrimental, as elevated *Ruminococcaceae* levels have been shown to be associated with myocardial infarction in humans and chronic cerebral ischemia in rats. A five-fold reduction in bacteria from the *Prevotellaceae* family could also be interpreted as a negative effect. Lower levels of *Prevotellaceae* have been linked to Parkinson’s disease and cognitive impairment induced by high-fat diets, whereas its high abundance correlated positively with enhanced resistance to ischemic injury. KD significantly reduced *Lachnospiraceae* levels, with a similar decrease associated with various brain pathologies. Similarly, KD increased *Oscillospirales* and decreased *Bacteroidaceae*, both previously associated with cerebral infarction in humans. Conversely, KD reduced *Burkholderiales*, which are associated with increased epilepsy risk, potentially explaining part of the diet’s efficacy in epilepsy patients. Research in humans showed that a 6-week KD increased the *Firmicutes/Bacteroidetes* ratio and decreased *Actinobacteria*, indicating dysbiosis (Akansel et al., 2024). Although the KD increased the abundance of *Oscilibacter*, *Blautia*, and *Akkermensia*, which are considered beneficial for metabolic health, in parallel, it led to a decrease in beneficial families such as *Prevotellaceae* and *Bifidobacterium*, while potentially pathogenic bacteria such as *Escherichia*, *Klebsiella*, and *Listeria* increased. In addition, it was found that fecal levels of short-chain fatty acids, which provide significant health benefits by promoting a healthy gut environment, reducing inflammation, modulating the immune system, and regulating metabolism, decreased after KD treatment, indicating adverse effects. Overall, the KD-induced changes in the gut microbiota show a dual character, as it simultaneously affects bacteria associated with beneficial effects and with pathological conditions.

Rapid restoration of energy balance is critical for stroke patients, and the long-term induction of ketosis limits the use of a ketogenic diet in the acute phase, as it takes several days to reach therapeutic concentrations of ketone bodies under KD. In addition, the KD has several contraindications and side effects that make it unsuitable for certain patient groups, particularly patients with liver disease, pancreatic insufficiency, gallbladder disease, and elderly patients. The KD can cause hypoglycemia, hyperlipidemia, electrolyte imbalance and dehydration, nausea, constipation, weight loss, increased cholesterol levels, and an increased incidence of infectious diseases. Right at the beginning of the KD, a condition known as “keto flu” may be observed, which is also usually considered a side effect. For most people, the symptoms of keto flu disappear within 1–4 weeks of starting the KD as the body becomes accustomed, and this phase can be alleviated by a gradual transition to the diet.

Exogenous ketone bodies appear to be safer and have fewer side effects, but they show considerable variability in neuroprotective effects. This ambiguity makes it difficult to develop standardized protocols for use in clinical practice. Although ketone bodies such as beta-hydroxybutyrate can cross the BBB, the extent of their penetration and concentration in neurons depends on many factors, including the individual characteristics of the patient’s metabolism and the state of the BBB during ischemic injury. In patients with an ischemic stroke, the permeability of the BBB may be impaired, which does not always lead to an improvement in the flow of ketone bodies. In addition, the activity of monocarboxylate transporters, which are responsible for the transport of ketone bodies across the BBB, often decreases in the ischemic zone. Therefore, even when ketone bodies are successfully transported to the brain, they are not always effectively taken up by the neurons and metabolized in the mitochondria.

When choosing a strategy for the treatment of acute injury, it should be considered that the use of ketone bodies outside the KD is associated with a different metabolic profile than in the KD. Ketone bodies are usually administered with a normal carbohydrate proportion and amino acids. Conversely, the KD is associated with carbohydrate restriction and leads to decreased aspartate production and increased glutamine synthesis, which is not observed when ketone bodies are administered. In addition, the KD significantly alters the composition of the gut microbiota in both animal studies and clinical trials, which is not the case with the use of ketone bodies. More detailed studies comparing the similarities and differences in gut microbiota changes during KD or ketone body administration are needed.

**Conclusion:** Thus, bypassing the mitochondrial respiratory chain with alternative metabolic substrates such as ketone bodies successfully modulates neural tissue metabolism, which could be useful for treating various types of injuries, including ischemic strokes, brain injuries, or other types of acute injuries. It should be noted that KD and its mimetics can have both positive and negative effects in stroke (**Figure 1**). In particular, in addition to a shift in metabolism and positive effects on mitochondria, KD may affect intracellular signaling pathways, the immune system, and the gut microbiota in unclear ways, which may antagonize neuroprotective mechanisms or even exacerbate the damage. In addition, it is important to consider that the increased levels of ketone bodies may be associated with several side effects, including problems with delivery to neurons. Finally, most patients with ischemic brain disease have multiple comorbidities (e.g., diabetes, cardiovascular disease) for which the ketogenic diet or infusion of ketone bodies may have an unpredictable effect. Large-scale randomized trials are needed to determine the efficacy and safety of ketogenic therapy in acute cerebral ischemia.

*This work was supported by the Russian Science Foundation, grant 24-75-10013 (to NVA).*
